# A Systematic Review of Healthcare Providers’ Approaches to Practices That Contribute to Secondary Victimization of Sexual Assault Survivors

**DOI:** 10.3390/healthcare14142111

**Published:** 2026-07-14

**Authors:** Ashley M. Ruiz, Julia F. Hammett, Stefani N. Baca-Atlas, Kaylen M. Moore, Jennifer Weitzel, Mitchell Kirwan, Lucy Mkandawire-Valhmu, Kaboni Gondwe

**Affiliations:** 1 Nell Hodgson Woodruff School of Nursing, Emory University, Atlanta, GA 30322, USA; 2 Edson College of Nursing and Health Innovation, Arizona State University, Phoenix, AZ 85004, USA; jhammet2@asu.edu; 3 Carolina Population Center, University of North Carolina at Chapel Hill, Chapel Hill, NC 27599, USA; sbaca-atlas@unc.edu; 4 College of Nursing, Marquette University, Milwaukee, WI 53233, USA; kaylen.moore@marquette.edu; 5 Wisconsin Local Health Departments, Sauk County Wisconsin, Baraboo, WI 53913, USA; jennifer.weitzel@saukcountywi.gov; 6 Department of Psychology, The University of Texas at El Paso, El Paso, TX 79968, USA; mekirwan@utep.edu; 7 School of Nursing, University of Minnesota, Minneapolis, MN 55455, USA; lmkandaw@umn.edu; 8 School of Nursing, University of Washington, Seattle, WA 98195, USA; kwgondwe@uw.edu

**Keywords:** secondary victimization, sexual assault, healthcare, violence prevention, women’s health

## Abstract

**Background/Objectives:** Secondary victimization (SV) following sexual assault (SA) is known to compound negative health outcomes for survivors seeking help within formal systems, such as healthcare. While there have been advances in preventing SV and support for SA survivors seeking help within healthcare, experiences of SV persist alongside worsening health disparities, particularly among disenfranchised populations of women. This literature review aims to examine the literature on HCP approaches to practices that may contribute to the SV of SA survivors. **Methods:** Following the PRISMA 2020 guidelines, the literature from three databases (PubMed, CINAHL Plus, and PsycINFO) was collected, followed by the screening of the titles, abstracts, and full texts of primary data sources available in full text and published in English between 2009 and 2022. The articles included for synthesis were appraised using the Johns Hopkins Nursing Evidence-Based Practice Model (JHNEBP), and the data extracted were analyzed using thematic analysis. **Results:** Four HCP approaches to practice were found to potentially contribute to the SV of SA survivors: (1) incomprehensive care in clinical practice; (2) a lack of collaboration between inter-agencies, healthcare organizations, interdisciplinary teams, and HCPs with SA survivors; (3) barriers to HCP training and preparedness to respond to SA; and (4) failures to implement trauma-informed care (TIC) frameworks. **Conclusions:** Based on these findings, we provide recommendations for the prevention and mitigation of the SV of SA survivors by integrating philosophical frameworks to guide healthcare practice, including person-centered care, trauma- and violence-informed care (TVIC), and cultural safety. We reflect on the current gaps identified from this systematic review and the need for future research to operationalize and measure SV after SA in healthcare.

## 1. Introduction

Healthcare responses are critical in supporting sexual assault (SA) survivors’ attainment of optimal health outcomes while simultaneously interrupting continuums of violence that often co-occur with SA [1,2,3]. Healthcare is a formal system that can recreate the victimization of SA survivors seeking help, a phenomenon known as secondary victimization (SV) [1,2,4]. Since healthcare institutions are inherently dependent on the practices of healthcare providers (HCPs) who respond to SA survivors, understanding approaches within HCP practices that may contribute to SV is critical for developing effective practices to prevent and mitigate added harm to SA survivors in healthcare delivery [1,2,4]. However, little is currently known about approaches to practice that may contribute to the SV of SA survivors by HCPs. This systematic literature review aims to address this gap. The implications of our findings may inform future directions in healthcare practice to prevent or mitigate SV in the delivery of care to SA survivors.

### 1.1. Secondary Victimization in Healthcare: Systemic Healthcare Responses to Sexual Assault

SV is defined as a type of violence that recreates social conditions encountered during SA within formal systemic responses [1,2,4]. SV entails separate and added experiences of victimization that control or limit survivors’ autonomy within formal systems, such as healthcare [1,2,4]. SV occurs from unresponsive treatments that compound and extend additional experiences of trauma to SA survivors [1]. SV in healthcare includes limiting survivors’ autonomy by impeding or denying available treatment options or opportunities to support survivors’ health needs [2,4,5].

SV is shown to result in poorer physical and psychological health outcomes for SA survivors [1,4,5]. Not only are survivors denied (or receive only limited) provision of timely or available options to support optimal health outcomes, but they also experience significantly higher levels of suicidality and emotional distress (including self-blame, anxiety, and social distrust) from SV during the provision of healthcare [4,5]. In consequence, SV in healthcare delays survivors’ recovery from SA and deters survivors from accessing healthcare that could support optimal health outcomes across the lifespan [1,4,5].

### 1.2. Trauma-Informed Care (TIC) and the Healthcare Response to Sexual Assault Nurse Examiners

In healthcare delivery, trauma-informed care (TIC) provides a holistic approach to guide HCP responses to survivors of traumatic experiences of security to optimize recovery [6]. This approach is informed by the integration of the following four key assumptions (4 Rs): realize (the pervasive, widespread impacts of trauma), recognize (screening and understanding the signs of trauma), response (prioritizing sensitivity to histories of trauma to promote healing), and resist re-traumatization (preventing triggers from prior trauma to build trust and safety) [7]. With the widespread pervasiveness of trauma, the implementation of TIC to guide HCP practices is recommended to be a universal precaution, in which all patients are assumed to have experienced trauma [7]. While TIC is endorsed as a gold standard in healthcare, efforts to implement and integrate TIC across healthcare and the healthcare workforce continue to vary [6].

Sexual Assault Nurse Examiners (SANEs), broadly known as Forensic Nurse Examiners, play a vital role in the coordinated response to SA and prevention of SV. SANEs are registered nurses who have completed specialized education and training to provide comprehensive TIC to SA survivors [8]. The role of SANE providers is to mitigate the immediate and long-term health consequences of SA by providing appropriate healthcare treatment, the accurate collection and preservation of forensic evidence, and legal support through expert testimony [8]. Despite the critical role of SANEs, many survivors encounter substantial barriers to identifying and accessing this specialized care.

In 2022, a law required a national directory of SANE locations, but the effort was unfunded, leaving no comprehensive resources available to help survivors find nearby forensic exam services [9]. SA survivors also face challenges related to the availability of SANE programs due to the insufficient number of SANEs to meet the scale of SA [10]. A national survey indicated substantial patterns of SANE programs closing due to funding interruptions, limited support from hospital leadership and emergency departments, and difficulty meeting legal and regulatory standards [11]. This is in conjunction with workforce challenges related to retention, stemming from high turnover due to burnout, vicarious trauma, and compassion fatigue [12]. With these barriers to identifying and accessing specialized SA care, SV persists as a concern that threatens to further compromise survivors’ health outcomes.

### 1.3. The Operationalization of Healthcare Providers (HCPs) and Approaches to Practice

In this review, HCPs are defined as formally trained individuals or trainees with specialized knowledge to provide healthcare services to promote optimal health outcomes. Examples include social workers, mental health workers, nurses, physicians, nursing students, and medical residents [13]. While the scope of HCP roles varies, approaches to practice refer to the intentional and unintentional manners in which HCPs know how to respond to SA survivors as a part of systemic healthcare responses. This systemic knowledge informs HCP approaches to practice through social behaviors used in responses to SA survivors [14]. HCP approaches to practice are a critical function of applying systemic responses to SA in healthcare [15,16].

Healthcare is a social determinant of health known to determine people’s health, illness, and death outcomes across individual and systemic levels [17,18]. HCP approaches to practice are recognized to be shaped by attitudes and beliefs that may contribute to the SV of SA survivors and compound health inequities and disparities [19,20]. Designed to eliminate health inequities within healthcare treatment, trauma-informed care (TIC) frameworks are often considered the gold standard and expected to be upheld by HCPs in practice. However, current understandings of TIC integrations within HCP practice-based approaches to prevent or mitigate SV across the healthcare workforce are limited.

### 1.4. The Systematic Literature Review

Since systemic healthcare delivery is a multifaceted process inherently built and dependent on HCP approaches to practice that respond to SA survivors, it is critical to understand HCP approaches to practice that may contribute to the SV of SA survivors across the healthcare workforce. The lead author of this paper heads a growing program of research focused on women’s experiences of post-SA SV in healthcare in connection to their race and gender identities. While we recognize that SA survivors’ experiences of SV in healthcare are also essential to understand, SV in healthcare is recognized in the literature as the outcome of the dyadic responses of HCPs in practice with SA survivors. However, the literature examining HCP approaches to practice that may contribute to SV is often limited to evaluating healthcare responses to SA by profession (i.e., medicine, nursing) or specialty settings (i.e., mental health, emergency department) [4,5,21,22]. This is the first review to synthesize and evaluate the literature on approaches to practice that may contribute to the SV of SA survivors across healthcare professions and specialty settings. These findings hold serious implications for improving and informing future interventions focused on preventing or mitigating SV to survivors within healthcare responses to SA.

This systematic literature review aims to answer the following question: What approaches to practice may HCPs use to contribute to the SV of SA survivors? A systematic review is an ideal mechanism for summarizing general knowledge, identifying gaps in knowledge where evidence may be lacking or inconclusive, and presenting evidence on potential areas for developing future interventions to improve outcomes related to SV [23].

## 2. Methods

This systematic review was conducted in accordance with the Preferred Reporting Items for Systematic Reviews and Meta-Analyses (PRISMA) 2020 guidelines [24]. A comprehensive search was performed using three databases, including PubMed on 5 August 2023, Cumulative Index to Nursing and Allied Health on 5 August 2023, and Allied Health Literature (CINAHL) Plus and PsycINFO on 5 August 2023. Articles had to be peer-reviewed publications of primary data sources in English and with full texts published between January 2009 and July 2022. The titles, abstracts, and full texts of the articles obtained were manually screened by two or more co-authors, following the inclusion and exclusion criteria detailed below. In the event of a conflict, an additional co-author was involved to reach a consensus on whether to include the article in the synthesis, based on the inclusion and exclusion criteria. The protocol for this review is registered under OSF Registries (registration DOI 10.17605/OSF.IO/PKH9V) and is publicly available.

Ultimately, 18 sources were included for synthesis and quality appraisal using the John Hopkins Nursing Evidence-Based Practice Model (JHNEBP) [25], which is commonly used to evaluate the quality of research applied across contexts of interprofessional collaborative practice [25,26]. Since this paper focuses on HCPs broadly, the JHNEBP provided a structured approach to considering the strengths and limitations of research translated into practice across interprofessional healthcare contexts.

### 2.1. Search Strategy

**Figure 1:**
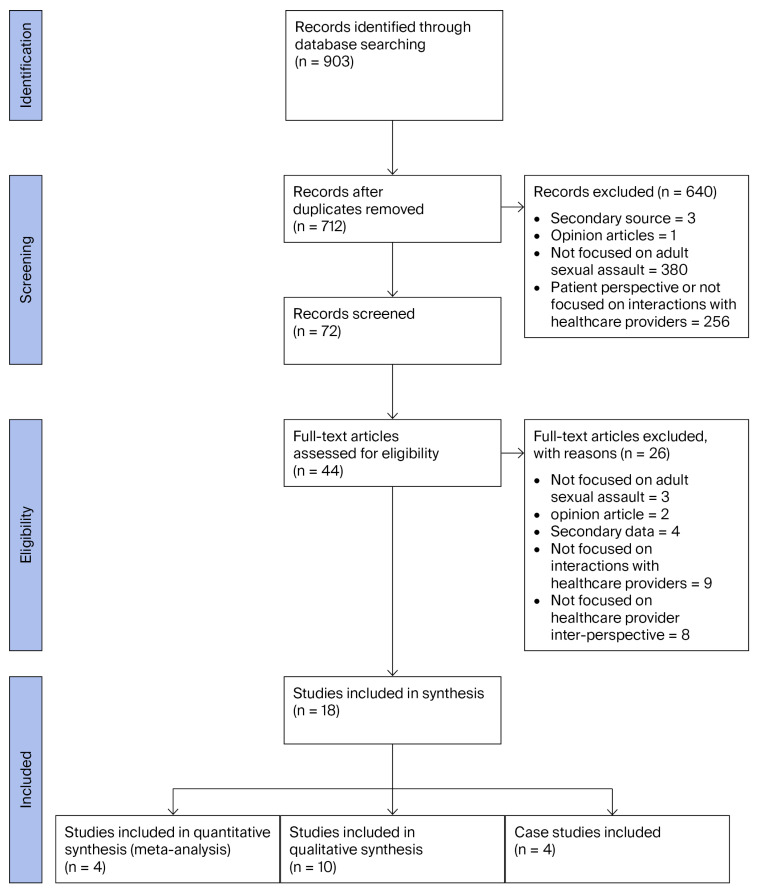
PRISMA 2020 flow diagram.

The databases searched with the number of corresponding results included PubMed, PsycINFO, and CINAHL Plus with Full-Text. The search terms used in each database included ((“Health Personnel” OR “Attitude of Health Personnel”) AND (“rape”) OR “Sexual Assault”) AND (second* victim*)) OR (“second rape” OR “second assault” OR “victim* blame*”) for PubMed; (“health personnel” OR “attitude of health personnel”) AND (“rape” OR “sexual assault”) AND (“second* victim*”) OR (“second rape” OR “second assault” OR “victim blame*”) for PsycINFO; and (“health personnel” OR “attitude of health personnel”) AND (“rape” OR “sexual assault”) AND “second* victim*” OR “second rape” OR “second assault” OR “victim blame*” for CINAHL. To improve accuracy by retrieving articles based on conceptual meaning rather than relying on exact word matches, MeSH (Medical Subject Headings) terms, which represent controlled, standardized vocabulary created by the National Library of Medicine to index the biomedical literature, were used in PubMed. PsycINFO utilizes MeSH-like controlled vocabulary structures, while CINAHL maps MeSH terms to its own CINAHL Headings. Full-text articles were further reviewed to assess eligibility specific to healthcare responses applied in practice that may contribute to the SV of SA survivors (Figure 1).

### 2.2. Inclusion and Exclusion Criteria

The inclusion criteria included (1) research articles published from January 2009 to July 2022, (2) English language, (3) studies assessing humans, and (4) peer-reviewed articles. The literature review search was conducted on 5 August 2023. Articles published in 2009 were included, as 2009 marked a global turning point in the emphasis on healthcare responses to SA, driven by the UN [27]. In addition, 2022 marked a global and cultural pivotal turning point related to the discourse of SA following the #MeToo Movement [28]. These global and cultural shifts related to SA involved global policy changes in healthcare responses to SA, including the reauthorization of the Violence Against Women Act (VAWA) in the United States, and global protections to protect reproductive health services following SA through the International Federation of Gynecology and Obstetrics [29,30]. For this reason, this literature review synthesizes the literature from 2009 to 2022. The participants of interest were HCPs, whose scope and formal roles were designed to support the treatment and health outcomes of SA survivors. In this paper, the authors focused on HCPs’ practices that may contribute to SV after SA. We considered articles focused on HCPs providing care to survivors of any gender identity. For the comprehensive inclusion of the literature focused on SA, we also included articles focused on rape, sexual violence, or violence against women. Since the operationalization of SV may differ across studies, articles that did not explicitly focus on SV, but rather used terms such as re-traumatization, second rape, victim-blaming, or harmful outcomes to SA survivors with healthcare delivery, were also included. To expand the applicability of the current review’s findings beyond the United States, any articles that were published in English (independent of whether the research was conducted within or outside of the United States) were included. This strategy was aimed at increasing contextual diversity by capturing evidence from diverse healthcare system responses to SA.

Articles were excluded if sources focused on HCP practices responding to child and adolescent sexual violence experiences. Sources were also excluded that focused on the SV of SA survivors outside formal healthcare system responses (such as law enforcement and education) and informal responses (such as public perceptions and media portrayals). Articles that focused on the re-traumatization of SA survivors perpetuated by informal sources of support, such as family members and friends of survivors, were excluded. Sources that focused on HCPs as secondary victims to crimes or as experiencing vicarious trauma were also excluded.

### 2.3. Study Selection

Initially, the titles and abstracts of sources were independently screened by two co-authors (Figure 1 PRISMA 2020 Flow Diagram). All articles with conflict in the title and screening phase were moved to full-text screening. In the event of conflict following full-text screening, a decision to retain or exclude the source involved an additional co-author to reach consensus.

Initially, conducting this search yielded 903 articles. Of the articles, 191 duplicate sources were removed, and the titles, abstracts, and full texts of 712 articles were screened for inter-rater reliability by a team of three reviewers. This screening involved the independent, blinded screening of the titles, abstracts, and full texts by two reviewers. The sources were then unblinded and compared by the two reviewers. If a conflict arose, a third independent reviewer was involved to determine whether to exclude or include the article. The overall percentage of agreement was high (greater than 0.95) among reviewers. In screening the titles and abstracts of articles, 640 articles were excluded, and 72 were screened. A total of 44 full-text articles were then eligible for synthesis, and 26 were excluded based on the established inclusion and exclusion criteria. Ultimately, 18 total sources were included for synthesis.

### 2.4. Study Quality and Risk of Bias Appraisal

The literature reviewed was critiqued using the JHNEBP. The JHNEBP model is designed to support the delivery of evidence-based practice (EBP) for nurses and, more broadly, healthcare providers [26]. As an inquiry-based framework, the JHNEBP model provides a structured approach for evaluating the quality of evidence across different study types to translate research into practice [26]. Table 1 summarizes the evidence quality criteria included in the JHNEBP across different study types. Utilizing the JHNEBP supported a structured approach to evaluating the strengths and limitations of different study types when synthesizing the literature on approaches to practice that may contribute to the SV of SA survivors. Inter-rater reliability was used among a team of three quality appraisers. Two appraisers independently evaluated selected studies included in the following screening using the JHNEBP. Two quality appraisers independently followed the JHNEBP model to categorize the level of evidence. The quality appraisers then compared appraisals through collaborative discussions, and in the event of a conflict, a third independent appraiser was involved to understand the rationale for the discrepancy and to determine a resolution. The majority of studies were of high or good quality, with one study of low quality. To provide a comprehensive summary of the current literature on approaches to HCPs’ practice that may contribute to the SV of SA survivors, all high- and good-quality studies were included, along with one low-quality study for synthesis. By including high- and good-quality studies with a low-quality study, this methodological approach helps prevent publication bias in study selection. In addition, the inclusion of high-, good-, and low-quality studies in this review provided valuable insight into SV across different healthcare systems and cultural taboos regarding SA.

In utilizing the JHNEBP, each included source was first evaluated to determine its level classification, which ranged from level I to level V. Levels I through III represent research-based evidence. The level of research-based evidence is determined based on the research methodology, whether the report includes a single study or multiple studies, and the research design. Level I studies are randomized controlled trials or experimental studies, level II studies are quasi-experimental, and level III studies are non-experimental. Qualitative studies that are a single research study or a meta-synthesis of multiple qualitative studies are considered level III evidence. Level IV studies include clinical practice guidelines or position statements, while level V evidence includes literature reviews, expert opinions, and case reports.

After determining each study’s classification in terms of level, sources were appraised to evaluate the quality of the evidence, as A (high quality), B (good quality), or C (low quality). The JHNEBP provides a set of criteria with guidelines to determine the quality of the evidence being appraised (see Supplementary Materials). An “A” (high-quality) designation requires that the evidence meets all or more than half of the criteria in detail. B (good-quality) evidence was determined by meeting half or less than half of the criteria in detail. C (low-quality) sources of evidence meet one or none of the established criteria in detail.

Using the JHNEBP tool, single qualitative studies are rated following a set of criteria. This initial set of criteria is then used to determine the quality rating based on transparency, diligence, verification, self-reflection and self-scrutiny, participant-driven inquiry, and insightful interpretations [25]. An overview is provided under Supplementary Materials for the required aspects for demonstrating transparency, diligence, verification, self-reflection and self-scrutiny, participant-driven inquiry, and insightful interpretation of single qualitative studies. Studies that demonstrated at least four aspects of the set of criteria were considered A (High quality). Studies that demonstrated three aspects of the criteria were rated B (Good quality), while studies demonstrating two or fewer criteria were rated C (Low quality). Table 2 provides details of the quality appraisal of synthesized studies using the JHNEBP.

### 2.5. Data Extraction

The process of data extraction was guided by a customized data extraction template in a shared electronic Word document. The data extracted included information about the article (including the author and year, funding, and institution), the methods (including the approach, study design, aim or hypothesis, and participant recruitment and selection), the outcomes (setting the time frame of the data collected and reported), and the results (themes identified, the intervention and measures used, and the participant sample obtained). Following the data extraction template, two co-authors independently and manually reviewed and extracted data from all the included sources. Afterward, the independently extracted data were compared by the two co-authors through a collaborative discussion. In the event of a conflict, an additional co-author was involved in an additional collaborative discussion to reach a consensus.

All the results extracted focused on different HCP approaches that may be associated with the outcomes of the SV of SA survivors in healthcare. The outcomes of the SV of SA survivors by HCPs included four broad domains and are reported with corresponding large frequency effect sizes as follows: impaired psychological or emotional well-being (78%), compromised social functioning with HCPs (78%), poor treatment within healthcare (89%), and occupational or professional consequences for HCPs (89%). A convergent synthesis approach was used in this systematic review to integrate the extracted qualitative and quantitative data to address the research question [49]. An overview of the qualitative results of the four quantitative studies included for thematic synthesis is provided in Table 3.

### 2.6. Data Synthesis

As mentioned, thematic analysis following guidelines by Braun and Clarke (2006) was used to synthesize findings in this systematic review [50]. Thematic analysis is a systematic process of identifying and interpreting patterns of meaning within data [50,51]. In this study, we utilized the five steps of thematic analysis, which include familiarization, creating an initial thematic framework, indexing and sorting, reviewing data extracts, and displaying data in summary [50,51].

In applying Braun and Clarke’s (2006) guidelines for thematic analysis, a team of three co-authors analyzed the extracted data using inter-coder reliability [50]. This entailed independent engagement in familiarization and the line-by-line open-pattern coding of recurring keywords, in which segments of text from extracted data were highlighted and initial insights documented [50,51,52]. Afterward, the three team members deliberated in group discussions to compare highlighted text and recurring keywords. This deliberation allowed for the comparison of open codes and the resolution of conflicts of recurring keywords identified to reach consensus on codes to include in a code book. This inductive process provided the basis for developing codes and initial descriptive themes based on recurring keywords in the extracted textual data [50,51,52].

After reaching consensus, the open-pattern codes based on recurring keywords were reorganized and refined to form the codebook with brief labels summarizing key ideas of the data [50,51]. The codes applied to the extracted data were indexed and sorted to illuminate patterns within the data to construct an initial thematic framework of approaches to HCP practices that may contribute to the SV of SA survivors by HCPs [50,51]. This entailed organizing codes into categories of patterns that may have contributed to the SV of SA survivors by HCPs [50,51,52]. These categories were then compiled into “piles” independently by three co-authors [50,51,52]. The three co-authors then engaged in a second group discussion to resolve discrepancies in the organization of categories into “piles” used to develop the initial themes. The themes of the initial thematic framework were summarized and reviewed again by three co-authors against the coded data to ensure consistent comparison with the themes and extracted data [50,51,52]. Discrepancies were resolved through a final collective discussion, and the finalized thematic framework was generated.

## 3. Results

### 3.1. Description of Studies

All 18 studies explored HCP practices that may contribute to the SV of SA survivors (see Table 3); Table 4 presents an overview of the results for each study analyzed. Six studies originated in the US, two in Kenya, and one each in the following countries: Canada, France, Italy, Cape Verde, Taiwan, India, Australia, Papua New Guinea, Trinidad, and Israel. Of the literature reviewed, 12 studies were published between 2018 and 2022, while six sources were published between 2010 and 2017 (see Table 3 and Table 4). HCPs included healthcare roles such as emergency medicine residents, doctors, social workers, SANE, mental health workers, and nursing students, as well as unspecified roles in healthcare that combined different functions within their sample (see Table 3). An overview of the synthesized study characteristics is presented in a matrix in Table 3.

### 3.2. Study Quality and Risk of Bias of Individual Studies

An overview of the quality appraisal using the JHNEBP tool, including considerations of bias within the synthesized individual articles, is available in Table 2. To summarize, the high-quality level III articles in this review include one quantitative study and seven single qualitative studies. The quantitative [31] and two of the qualitative [33,34] studies included HCPs’ experiences of interpersonal collaboration within community, healthcare, and legal systems designed to support SA survivors. Tien (2017) [31] proposed a one-stop service to avoid SV and to improve the delivery of quality forensic examination and prosecution of sexual violence. Notably, despite a sufficient sample size, the findings of Tien’s (2017) [31] study should not be interpreted as implying causality given its cross-sectional design. Greeson (2018) [33] examined SART leaders’ beliefs about different aspects within local sociocultural communities that influenced the effective use of SARTs. This study included a representative sample of 169 SART members, but the majority were SART leaders from rural rather than urban or suburban communities [33]. Kelty (2018) [34] clearly stated the purpose and aim of the study to explore responses among professional groups (including HCPs) regarding effective investigations and trial prosecutions of SA. However, details of strategies to enhance trustworthiness, such as member checking, were not discussed.

Three high-quality level III single qualitative studies (Goldblatt et al., 2022; Munala et al., 2018; Munala et al., 2022) explored HCPs’ experiences in responding to survivors of SA in practice [32,36,37]. For instance, Goldblatt (2022) [32] examined HCPs’ motives and processes for addressing SA against women in later life. Munala et al. (2018) [37] and Munala et al. (2022) [36] published results from the same study, which aimed to explore the perspectives and experiences of HCPs that provide care to female rape survivors.

The remaining two high-quality level III studies include qualitative studies by Kirkner et al. (2021) and Patterson et al. (2020) [35,38]. These two studies differ from the other high-quality level III studies in their focus, as one examined patient-centered orientation through educational training among HCPs specific to the care of SA survivors [38], and the other examined experiences among survivors and survivor/support provider dyads regarding formal and informal responses to survivors disclosing SA [35].

The high-quality level V studies in this review include four unique case studies that lack generalizability but provide expert recommendations. Specifically, these studies include two case reports focused on patients with severe injuries from nonconsensual fisting [40,42] and two case reports focused on patients at the intersection of SA and HIV [39,41]. Recommendations are aimed at providing beneficial interventions, while avoiding re-traumatization and stigmatization, to prevent added harm (i.e., SV) to SA survivors. Two quantitative, good-quality level II studies focused on the education of resident physicians [42] and medical students [44]. The study by Auten et al. (2015) utilized a small sample size [43]. In contrast, the second study by Franchitto and Rougé, 2010, appraised legal-medicine education with an adequate sample size; however, the results cannot be generalized beyond the French context [44].

The good-quality level III studies include one quantitative [47] and two qualitative studies [45,46]. The quantitative cross-sectional study by Strunk (2017) utilizes a sufficient sample size to examine pre-nursing and baccalaureate nursing students’ knowledge, attitudes, and beliefs about SA [47]. However, the generalizability of these findings is limited by the predominantly White, female sample from a large Midwestern university [47]. Similarly, Silva et al. (2021) clearly stated the purpose of the study to explore Primary Healthcare workers’ perceptions of violence against women [46]. However, they did not present a justification for the method used or strategies to support the trustworthiness of the data interpretations. Finally, Nathaniel (2021) explored social workers’ views of intimate partner violence in Trinidad [45]. The sample size, participant characteristics, and process used to analyze and interpret the data are not presented [45].

The final study by Bhagat et al. (2018) presents definitive recommendations to prevent revictimization or SV, but it was rated a low-quality level III source [48]. This study failed to present the instruments used in the questionnaire or the rate of response to the distributed questionnaire, or to consider the study’s limitations [48].

### 3.3. Summary of Findings

Using thematic analysis, four approaches to practice may contribute to the SV of SA survivors by HCPs: (1) incomprehensive care in clinical practice; (2) a lack of collaboration between inter-agencies, healthcare organizations, interdisciplinary teams, and HCPs with SA survivors; (3) barriers to HCP training and preparedness to respond to SA; and (4) deviations in the implementation of trauma-informed care (TIC) frameworks.

1. Incomprehensive Care in Clinical Practice

Incomprehensive care in clinical practice entailed HCP approaches that inadequately addressed SA survivors’ full human health needs, resulting in separate and additional harm to or SV of survivors [37,38,39,41,42]. Of the 18 studies analyzed, 15 reported that the provision of incomprehensive care by HCPs may be associated with SV among SA survivors [32,33,34,35,36,37,38,39,41,42,45,46,47]. Studies described incomprehensive care provided by HCPs in clinical practice that inadequately addressed the psychosocial dimensions of health needs and resulted in added burden or harm to SA survivors [32,33,34,35,36,37,38,39,41,42,45,46,47].

Among these studies, internalized disbeliefs of SA by HCPs extended into clinical practice as incomprehensive care in responding to SA survivors. HCPs did not believe SA survivors about occurrences of SA, as well as not believing reports of SA occurring within an institution, population, or community [32,33,34,35,36,37,41,46,47]. Clinical HCP practices that silenced SA survivors included not believing SA survivors, as well as HCP responses of disgust, repulsion, or shock to disclosure [32,33,35]. As a result of these misconceptions, SA survivors were denied opportunities to discuss and process the trauma of SA within HCP responses [32,35,38,39,42]. Occurrences of not believing reports of SA were informed by rape myth beliefs (RMB) and victim-blaming within HCPs’ approach to practice [32,33,34,35,36,37,41,46,47].

Victim-blaming in clinical practices stemmed from RMBS within HCP practices that failed to provide comprehensive care. Nine studies identify RMBs and victim-blaming within HCP practices as a critical component of the provision of incomprehensive care by failing to attend to the psychosocial needs of SA survivors [32,33,34,35,36,37,41,46,47]. RMBs are false beliefs about SA, survivors of SA, and perpetrators of SA [33,34,47]. RMBs often place the blame for the SA onto survivors rather than the perpetrator; this is also known as victim-blaming [33,34,35,36,37,45,47]. In this review, RMBs and victim-blaming within HCP practices include judging the legitimacy of SA survivors, denying that SA occurs within a community, and believing that only pathological behavior explains the perpetuation of SA [32,33,34,35,36,37,41,46,47].

Victim blaming within HCP practices also resulted from social misconceptions that are specific to populations (based on gender, age, race or ethnicity, occupation, mental health, socioeconomic status, and context) within practices used by HCPs [32,33,36,37,38,41,42,45,46,47]. A majority of studies reported on the detrimental impact of gender misconceptions of women and context within HCP practices that may contribute to the SV of SA survivors [32,33,37,38,42,45,46]. Four studies describe the SV of SA survivors due to HCPs’ perception of normal trauma responses (such as agitation, fatigue, and memory recollection) as socially disruptive behavior or a sign of being “weak” [36.38,50]. These misconceptions within HCP practices were associated with added psychosocial harm by silencing and stigmatizing SA survivors [32,33,36,37,38,39,42,45,46,47].

Finally, five sources reported a lack of therapeutic communication within HCP approaches to practice, which may contribute to SV [32,35,38,39,42]. Non-therapeutic communication encompasses not demonstrating empathy, validation, or active listening; failing to challenge survivors’ self-blame; asking unnecessary questions; or not allowing space for survivors to tell the story of their trauma [32,35,38,39,42].

The psychosocial harm resulting from incomprehensive care denied SA survivors access to clinical treatment opportunities [34,36,39,41,42]. This includes failing to provide options (including emergency contraception, treatment for sexually transmitted infection, and HIV counseling and testing following SA), as well as failure to provide forensic exams or not gathering viable evidence specimens for investigation [34,36,39,41,42]. The provision of incomprehensive care also included HCP practices that prioritized clinical tasks (such as DNA or evidence collection) over the psychosocial treatment needs of the SA survivor [34,36,38].

2. Lack of collaboration between inter-agencies, healthcare organizations, interdisciplinary teams, and HCPs with SA survivors

In this review, 12 studies discuss a lack of collaboration between inter-agencies, healthcare organizations, interdisciplinary teams, and HCPs with SA survivors that may have contributed to SV [31,33,34,35,36,37,39,40,41,42,45]. A lack of collaboration involved limiting available resources to support the attainment of SA survivors’ health goals [31,33,34,35,36,37,39,40,41,42,45]. Of the studies included for analysis, 12 studies discuss a lack of collaboration between inter-agencies, interdisciplinary teams, and HCPs with SA survivors that may have contributed to SV [31,33,34,35,36,37,39,40,41,42,45].

Eight studies discuss a lack of inter-agency collaboration among multiple organizations, including private and government sectors (police, legal, health, and forensic science), that contributes to the SV of SA survivors by HCPs [33,34,36,37,40,41,42,45]. A critical barrier to inter-agency collaboration was a lack of buy-in and underfunding of hospitals, healthcare facilities, and staff involved in coordinating inter-agency resources and decisions [33,34,40,41,42]. This included equipment to support complete documentation, collect forensic evidence (through digital photography, colposcopy with nuclear staining, and anoscopy), screen and treat preventable or manageable conditions, and provide expert witness testimony [33,34,36,37,40]. As a result, SA survivors received delayed HCP responses, inaction, and time-consuming procedures that limited SA survivors’ attainment of optimal health outcomes [31,41,45].

The lack of buy-in or funding also impacted a lack of collaboration with SA survivors from healthcare organizations [36,37,42,45]. Although protocols and guidelines are often established within healthcare organizations to guide practices, applying protocols and guidelines to daily HCP practices was not always possible due to a lack of resources, high patient volumes, and time constraints [36,37,42,45]. In turn, three studies found that HCPs differed from protocols and guidelines; this may have contributed to HCPs making judgmental decisions to determine the legitimacy of an SA survivor based on the SA survivor’s presentation [36,42,45]. Another study discussed HCPs’ secondary trauma in navigating the gaps between HCP practices and policies to best support SA survivors due to family interference [36].

The lack of collaboration was detrimental to the utilization of interdisciplinary teams (such as forensic medicine specialties and multidisciplinary sexual assault response teams (SARTs)) to support SA survivors’ health goals [33,34,36,40,42]. For instance, one study included in this review found that forensic nursing and medicine specialties were intentionally and unintentionally semi-invisible [34]. Ten studies in this review found a lack of collaboration from HCPs with SA survivors [34,35,36,37,39,40,41,42,45]. This included excluding SA survivors from making decisions, HCPs’ failures to offer safe and accessible options, and/or not being provided information to facilitate SA survivors’ ability to make informed decisions about their own healthcare [35,39,42,45].

The lack of collaboration from HCPs with SA survivors may have contributed to SV having serious short- and long-term consequences for survivors’ economic status and safety beyond seeking healthcare [35,36,37,39,41,42,45]. For example, sources in this review discuss the economic impact of seeking healthcare (from traveling long distances for healthcare to the delayed treatment of conditions) on SA survivors’ economic status, including relationship, housing, and education stability, and household dynamics [35,36,37,39,41,42,45].

3. Barriers to HCP training and preparedness to respond to SA

Barriers to HCP training and preparedness to respond to SA included HCP practices that resulted in the SV of SA survivors due to misinformed, unfamiliar, or inadequate training in the provision of healthcare following SA [37,38,43,44,48]. The studies in this review found that providers were often unaware of policies and resources available to guide care and lacked education or training about SA. For example, one study found that 36.7% of physicians were unaware of guidelines and protocols for medicolegal care, 58.2% of physicians were unaware of the legal age of consent in relation to jurisdictional definitions of SA, and 61.2% had never received education or training on SA [48].

Similarly, another study found that, although support materials were available, 70.4% of medical students reported not using materials, and 20.5% of students did not know they existed [44]. In this study, 92.4% of medical students in their third to sixth year of medical school felt unprepared to examine survivors of assault because they felt inadequate in their knowledge of the law and risks associated with medical practice. HCPs’ lack of training to support their own internal coping in response to disclosures from SA survivors also contributed to experiences of SV perpetuated by HCPs [32,35,37].

For example, in one study included in this review, providers discussed their lack of readiness, which may have contributed to reactions of shock, speechlessness, and a general loss of language, creating a barrier between HCPs and survivors of SA [32]. This emotional incapacitation due to unpreparedness may have contributed to providers reacting to disclosures with a sense of being “infected” afterward, or “opening a Pandora’s box” [32]. Such reactions could explain providers’ overall hesitation in addressing survivors’ healthcare needs following SA. Similarly, another study included in this review, which analyzed recommendations for responding to SA survivors from the perspective of both informal support persons (i.e., friends, family members, significant others) and survivors, found that informal support persons articulated the importance of HCPs engaging in self-care when coping with the emotional weight of disclosure [35].

4. Failures to Implement Trauma-Informed Care (TIC) Framework

Failures to implement the TIC framework by HCPs in practice may also contribute to the outcomes of the SV of SA survivors [32,35,36,37,38,39,42,45,46,47]. In this review, twelve studies describe failures in the implementation of TIC in practice by HCPs that may contribute to the SV of SA survivors [32,35,36,37,38,39,42,45,46,47].

Two studies explicitly named TIC frameworks’ failure within HCP practices that impact the SV of SA survivors [39,42]; seven studies describe HCP practices that deviate from TIC frameworks that may contribute to the SV of SA survivors [32,35,36,37,38,46,47]. For instance, in this review, four studies discuss HCPs’ misconceptions about neurobiological trauma responses to memory recall and retrieval, which may be associated with SV in SA survivors [32,35,36,42].

The four studies discuss HCPs’ misconceptions of trauma responses related to memory within HCP practices that judged the legitimacy of the SA due to survivors’ inability or difficulty retrieving and recalling detailed memories consistently [32,35,36,42]. This judgment encountered within HCP practices hindered SA survivors’ recovery [32,35,42]. As a result, survivors internalized a self-image of being untrustworthy and negative emotions (such as self-blame, shame, and helplessness) connected to aspects of their identity [32,35,36,42].

The inability to appropriately implement TIC frameworks often involves the underestimation of the risk and pervasiveness of SA by HCPs, which may contribute to the SV of survivors [32,36,37,39,42,44,45]. The underestimation of SA within HCP practices resulted in the delay of time-sensitive treatments (such as STIs, HIV, emergency contraception, and evidence collection). The substandard care received deterred survivors from accessing resources in healthcare, as well as other institutions in the future [32,36,37,39,40,42,44,45]. As a result, SA survivors hesitated in accessing potential resources within healthcare and other institutions, unless the violence became severe or fatal due to the anticipation of receiving inadequate care [40,42]. Consequently, the care rendered based on underestimations of SA within practices used by HCPs further compounded the social and economic losses that survivors incurred from SA, which may contribute to the outcomes of SV among SA survivors [32,35,37,39,42,45].

## 4. Discussion

Although healthcare has undergone cultural shifts to improve responses to SA in recent decades, the SV of SA survivors through HCP approaches to practices continues within present-day responses [5]. In this review, we identified four different approaches that may contribute to the SV of SA survivors due to HCP practices. These four approaches include (1) incomprehensive care in clinical practice; (2) a lack of collaboration between inter-agencies, healthcare organizations, interdisciplinary teams, and HCPs with SA survivors; (3) barriers to HCP training and preparedness to respond; and (4) failures to implement TIC by HCPs that may contribute to the SV of SA survivors. The findings of this review show that the SV of SA survivors due to approaches to practice used by HCPs does not occur in isolation [32,33,36,37,38,42,45,46,47]. Rather, the SV of SA survivors due to HCP practices is an extension of larger socioeconomic and political contexts that are ever-changing [32,33,36,37,38,42,45,46,47,48,53,54]. This shifting context shapes the social conditions within HCP approaches to practice, and the consequent risk of the SV of SA survivors in the provision of healthcare across systemic levels (i.e., micro-, meso-, and macro- levels of healthcare) [53,54].

At the micro-level of healthcare, between SA survivors and HCPs, our findings describe existing pathways through which SA is normalized within HCPs’ clinical practices that may be associated with the outcomes of the SV of SA survivors. These clinical practices recreate social conditions that devalue SA survivors, while also adding to issues of social stratification through controlling and limiting opportunities for SA survivors to access and receive quality healthcare. In this review, quality healthcare requires comprehensive care, which fundamentally entails a relationship that recognizes and responds to the full multidimensionality of human health (including biological, psychological, spiritual, environmental, and social dimensions) [55,56]. Of the studies included in this review, clinical practices of incomprehensive care occurred within the psychosocial dimensions of health, thereby negating SA survivors’ full dimensions of human health. These micro-level negations of full humanness are a form of epistemic injustice supported through internalized misconceptions and beliefs related to SA, as well as disenfranchised populations disproportionately vulnerable to SA within HCP clinical practices.

The prevalence of the trauma, stress, and adversities of SA, and subsequent health disparities, has been documented to be disproportionately high among historically disenfranchised populations, particularly racial minoritized women [57,58]. Disenfranchised populations include, but are not limited to, minoritized populations based on gender, race, economic status, age, and abilities [57,58]. The literature synthesized in this review recognized SA survivors of disenfranchised populations to be most at risk of SV due to the social conditions conducive to epistemic injustices within healthcare [32,33,36,37,38,40,42,45,46,47]. Epistemic injustices unfairly question SA survivors’ credibility or deny survivors’ experiences and ability to make sense of their experience in healthcare [59,60]. In this review, studies report the anticipation of SV in HCP clinical practice among SA survivors from disenfranchised populations [40,42]. This is similar to an existing study, in which survivors of disenfranchised populations are likely to conceal their experiences of SA to prevent SV by HCPs within clinical practice [60].

SA rarely occurs in isolation from other forms of violence across the continuum of sexual violence, which includes all sexual acts causing harm [1,61]. Meanwhile, the literature shows that the incidence of SA increases one’s risk for future victimization from SA as well as different types of aggression throughout one’s life course [1,62]. The disproportionate pervasiveness and prevalence of SA among women is recognized to be upheld by social processes of normalization [1]. In recent contemporary times, the systemic macro-levels of normalization within healthcare have contributed to healthcare being referred to as “reluctant partners” in providing healthcare services to SA survivors [63]. Within the literature, this reluctance is evident in healthcare policies that often prioritize evidence collection for legal purposes rather than delivering quality healthcare services to support survivors in attaining optimal health outcomes [60,63].

Our findings in this review highlight the challenges posed by a lack of buy-in to foster collaboration among healthcare organizations, agencies, and disciplines, which may contribute to SV outcomes for SA survivors. In this review, the lack of buy-in within larger systemic macro-levels of healthcare fosters social conditions of disjointed healthcare systems and unattainable daily healthcare workflows that add to burnout among HCPs, even when policy and guidelines are established [33,34,36,40,42]. The described reluctance of healthcare to provide healthcare services to SA survivors has been argued to be evident in healthcare organization policies that often prioritize evidence collection for legal purposes over the delivery of quality healthcare services to support survivors in attaining optimal health outcomes [60,63]. The result creates a healthcare culture in which HCPs sometimes view the delivery of healthcare to SA survivors as a burden that prevents them from completing “their ‘real’ or ‘urgent’ work of caring for the sick” [58,60]. In this review, these findings complement the literature by demonstrating the underlying macro-socioeconomic healthcare structures that fail to foster collaboration across meso-systemic or organizational levels of healthcare.

In conjunction, these underlying macro-socioeconomic factors may impede HCPs’ opportunities to attain adequate training to develop responses that prevent SV in SA survivors across practice trajectories. Of the studies that demonstrate a lack of training and preparedness to respond to SA, which may contribute to SV, two studies examined resident physicians, while three studies focused on HCPs with completed formal training [36,38,43,44,46]. Of the studies focused on HCPs with completed formal training, only one examined HCPs with completed formal sexual assault forensic examiner (SAFE) didactic training [38]. These studies describe a lack of familiarity, awareness, and comfort in responding to SA, while the findings of Patterson et al. (2020) reveal that SAFE training shifted HCPs’ views of their role in supporting survivors’ health needs [36,38,43,44,48]. In turn, this philosophical shift supported better emotional health outcomes and HCPs’ confidence in responding to and preventing the SV of SA survivors through SAFE training [35]. These findings are similar to those in the literature, in which SAFE training has been shown to significantly improve HCPs’ confidence in preventing the SV of SA survivors from 33% prior to SAFE training to 80% post-SAFE training [64].

Although TIC is recommended for universal use in healthcare practice, our findings highlight failures in its implementation, particularly in underestimating the pervasiveness of SA and understanding trauma responses [32,35,36,37,38,39,42,45,46,47]. In consequence, these failures to implement TIC may compound socioeconomic losses and contribute to the SV of SA survivors. The literature estimates that 79–84% of SA survivors do not seek healthcare immediately following an assault [65,66]. This is reported to be due to economic barriers to accessing healthcare, as well as difficulties tolerating aspects of healthcare delivery, compared with those without histories of SA [67]. However, it is important to note that the degree of socioeconomic barriers and losses that may contribute to the SV of SA survivors likely varies among differing socioeconomic contexts and subsequent, differing healthcare system models. In this review, the studies that describe the socioeconomic losses from failures to implement TIC by HCPs were conducted in the following corresponding countries: the United States, Cape Verde, Trinidad, Kenya, Israel, and Canada [32,35,36,37,38,39,42,45,46,47,48]. In consideration of these findings discussed, the following section presents implications for informing healthcare practices to prevent SV among SA survivors.

### 4.1. Implications for Healthcare Practices Guided by Philosophical Paradigm Shifts to Prevent SV of SA Survivors

Based on the results of this review, the prevention and mitigation of SV will require a multi-method approach centered on philosophical paradigm shifts from person-centered care to guide healthcare practices. For SA survivors, this philosophical shift requires centering the knowledge that SA survivors share about the challenges they face and about which solutions will be meaningful and sustainable within healthcare practice, as well as healthcare policy and research [68]. HCPs are often challenged in responding to the totality of a person within a predetermined systemic response that may normalize SA and contribute to the SV of SA survivors [34,36,38,39,40,41,42,43]. While patient-centered care focuses on attending to a person’s immediate health needs, person-centered care provides a broader approach to understanding an individual beyond the role of a “patient”; their immediate health needs; and in relation to their whole being, life course, and social context [69].

Considering the disproportionate prevalence of SA across the lifespan, particularly for women, an authentic paradigm shift in person-centered care provides a comprehensive understanding of human health across the lifespan that could support the prevention or mitigation of the SV of SA survivors in healthcare. Person-centered care is a global movement that calls for recognizing the inherent relational nature of healthcare delivery [70]. With this understanding, person-centered care prioritizes psychosocial human health, including people’s experiences within their context. With a philosophical shift to person-centered care, approaches to healthcare practice to prevent SV can be guided by a comprehensive understanding of health and by consideration of the psychosocial practices that may contribute to SV immediately following SA, as well as across a person’s life course. In addition, implementation studies evaluating the outcomes of person-centered care in healthcare have found improvements in collaboration between patients and providers, as well as improved HCP retention, communication, and HCP well-being [70].

While person-centered care offers valuable approaches for guiding healthcare practice in preventing SV among SA survivors, it does not critically examine ongoing power differentials, particularly within systemic healthcare responses to SA. However, two existing frameworks developed to guide cultural paradigm shifts in understanding power differentials in healthcare are trauma- and violence-informed care (TVIC) and cultural safety. TVIC builds on the principles of TIC and calls for recognizing the ongoing effects of structural violence in shaping people, their behavior, and their health [71]. With the integration of TVIC, the prevention and mitigation of the SV of SA survivors engage larger meso- and macro-systemic levels of healthcare decision makers in fostering structures and policies informed by an understanding of ongoing violence and trauma outcomes. TVIC is often used alongside cultural safety to support the delivery of safe, accessible, patient-centered care [71]. Cultural safety differs from person- and patient-centered care, and TVIC, in that patients are centered as active participants in their care and as the sole source for determining whether the care they received was safe [72]. In this way, cultural safety is a retort or adaptation within existing healthcare systems to the notion that HCPs are culturally competent through their training and education [72].

While person-centered care, TVIC, and cultural safety provide philosophical frameworks for guiding HCPs’ practices to prevent or mitigate SV among SA survivors, translating these frameworks across healthcare system models will require collective, systemic healthcare commitments to tailor effective implementation to specific contexts and populations.

### 4.2. Methodological Limitations of This Review

Although this review offers several strengths, it is not without limitations. While the overarching aim of this study was to compose a succinct yet comprehensive summary of the current literature on approaches to HCP practices that contribute to the SV of SA survivors, our search strategies and eligibility criteria may have prevented us from assessing additional information. For example, by focusing our search only on the most relevant databases (PubMed, CINAHL Plus, and PsycINFO), we may have excluded broader databases (such as Scopus, EMBASE, or Web of Science), which could have prevented us from discovering additional research. Moreover, we included only studies published in English, which limits our ability to incorporate articles from non-Anglo-Saxon contexts. This exclusion may restrict the generalizability of this review to healthcare systems in emerging economies or cultures with different taboos regarding SA. Similarly, to ensure quality control, we excluded non-peer-reviewed articles, which may have included additional information on the topic of interest.

### 4.3. Broader Limitations and Future Direction

Because all of the articles included in this review focused specifically on HCPs’ approaches to practice with SA survivors, it is unclear whether the current findings would be transferable to survivors of other forms of violence. SA often overlaps with other types of violence, such as intimate partner violence and sex trafficking [1]. Some of the individuals who took part in these studies and were included in this review may have experienced more than one form of intentional violence, which may have impacted their experiences with HCPs. Teasing apart whether—and if so, to what extent—the overlap of different forms of violence is associated with HCP responses to SA survivors and their risk for SV is important to appropriately tailor practice recommendations. Future reviews could focus on HCPs’ approaches to practices that contribute to the SV of SA survivors, as well as survivors with experiences of different forms of intentional violence.

Additionally, these findings pertain to HCP approaches to practices that may contribute to SV that involve the disclosure of SA to HCPs. However, many SA survivors may seek care without disclosing that they have experienced SA. These survivors may nevertheless be at risk for SV. The literature in this review does not reflect practices used by HCPs that contribute to SV among SA survivors who do not disclose SA, possibly resulting in biased findings. It is possible that survivors not disclosing SA to HCPs fundamentally differ from disclosing survivors, and, as a result, their experiences of SV may differ. Similarly, some of the articles in this review focused on HCPs’ perspectives on approaches to practices that may contribute to SV in SA survivors. HCP perceptions on approaches to practices that may contribute to SV may differ from those of SA survivors. Future studies that assess both providers and patients are needed to tease apart this overlap, or lack thereof.

Finally, it is important to note that, in most of the articles we reviewed, SV was not clearly operationalized or directly measured. Therefore, we inferred SV from findings related to victim-blaming, lack of training, poor communication, or inadequate care. Because SV was rarely measured as a distinct outcome in the primary studies, conclusions about practices that contribute to SV are in part based on interpretation. Future research is needed to clearly operationalize and measure SV among HCPs; we hope that the current review will inspire such work.

## 5. Conclusions

Although healthcare has undergone cultural shifts in recent decades to improve HCP approaches to practices that respond to SA survivors, the SV of SA survivors continues within healthcare. This literature review identified four HCP approaches to practices that may be contributing factors. These approaches to practices used by HCPs compound existing harm from SA and may add to pre-existing health disparities, particularly among disenfranchised populations of women, within systemic healthcare responses to SA. These findings underscore the importance of effectively implementing philosophical paradigm shifts to guide healthcare practices through the integration of person-centered care, TVIC, and cultural safety to prevent the SV of SA survivors. However, effective implementation will require a collective commitment to translating implementation tailored to specific populations and contexts. In addition, this review considers the limitations in connection with future directions. From this review, we reflect on the current lack of studies that directly measure SV in healthcare responses to SA and the future need to improve the operationalization and measurement of SV in healthcare after SA.

## Figures and Tables

**Table 1:** Quality of evidence criteria.

Type of Evidence	Low Quality	Good Quality	High Quality
Quantitative	Little evidence with inconsistent results; insufficient sample size for the study design; and conclusions cannot be drawn.	Reasonably consistent results; sufficient sample size for the study design; some control; fairly definitive conclusions; reasonably consistent recommendations based on a fairly comprehensive literature review that includes some reference to scientific evidence.	Consistent, generalizable results; sufficient sample size for the study design; adequate control; definitive conclusions; consistent recommendations based on a comprehensive literature review that includes thorough reference to scientific evidence.
Qualitative	The topic and aim of the review are not well defined. Literature search methods lack clarity and may or may not be appropriate. Literature synthesis is insufficient. Findings are not sufficiently linked to data analysis. Definitive conclusions cannot be drawn.	The topic and aim of the review are clearly stated. Literature search methods are adequate. Data analysis described. The literature was reasonably synthesized to achieve deeper understanding. Findings are linked to data analysis. Fairly definitive conclusions can be drawn.	The topic and aim of the review are clearly stated. Literature search methods are clear and appropriate. Data analysis is well-described. The literature is thoroughly synthesized to generate a deeper understanding. Findings are thoroughly linked to data analysis. Definitive conclusions can be drawn.
Mixed Methods	Contains good-to-low-quality quantitative and qualitative study components; study design not relevant to research questions or objectives; poorly integrated data or results; and no consideration of limits of integration.	Contains good-quality quantitative and qualitative study components; relevant study design; moderately relevant integration of data or results; and some discussion of limitations of integration.	Contains high-to-good-quality quantitative and qualitative study components; highly relevant study design; relevant integration of data or results; and careful consideration of the limitations of the chosen approach.
Case Study	Expertise is not discernible or dubious; conclusions cannot be drawn.	Expertise appears to be credible, draws fairly definitive conclusions, and provides a logical argument for opinions.	Expertise is clearly evident, draws definitive conclusions, and provides scientific rationale; thought leader in the field.
Single Qualitative Studies		Transparency: documentation of how researchers justified decisions, and collectively review the data with others, and delineate the process for creating themes and categories. Diligence: Reviews the data multiple times to confirm interpretations; seeks opportunities to engage with a multitude of sources to corroborate evidence. Verification: Ensures that the process aligns with methodological approaches. Self-reflection and self-scrutiny: The researcher engages continuously in how their own experiences, background, or prejudices may impact the analysis and interpretation. Participant-driven inquiry: Scope and breadth of questions in studies are shaped by participants; the analysis and interpretation of the data center the voices of participants. Insightful interpretation: The data and knowledge presented are connected in meaningful ways to the relevant literature.	

Reference: [26].

**Table 2:** Quality appraisal using the John Hopkins Nursing Evidence-Based Practice Tool.

Author/Year	Evidence Level (Quality Rating)	Assessment	Risk of Bias	Approaches to Practice and SV of SA Survivor Considerations
Tien/2017 [31]	Level III (A)	-Results: Consistent with limited generalizability. -Sample size: Sufficient for MANOVA and multiple linear regression. -Conclusions: Definitive conclusions are drawn based on the results of the analysis. However, limitations are associated with study design. -Recommendations: Consistent and informed by a comprehensive literature review on factors that contribute to interprofessional collaboration.	Results: Generalizability limited by convenience sample consisting mostly of women working in interprofessional settings in Taiwanese hospitals. Conclusions: Cannot claim causality of factors examined that impact collaboration for this cross-sectional study design.	-SV not directly measured or operationalized. -Authors indicate that one-stop service model was developed to prevent SV by improving the quality of forensic examination and prosecution rates.
Goldblatt/2022 [32]	Level III (A)	-The research question and phenomenon are clearly stated: “How do welfare and health care professionals present the motives and processes that obstruct exploring, exposing, and intervening in cases of sexual assault against women in later life (SAWLL)?” (Goldblatt, 2022 [32], p. 2751). -Justification of the method is clearly stated to understand the meaning of how people experience SAWLL. -The study sample is representative of healthcare professionals, including nurses, physicians, and social workers. -Researchers have experience and expertise in nursing, social work, and health sciences at the forefront of elder abuse and neglect. -Participant characteristics are described in terms of profession, gender, and professional experience. -Sample size is adequate for reaching data saturation. -Processes of verification through member checking and confirmation with participants are discussed. -Manual management of analysis is described. -Thematic findings are consistent with quotes from narrative data. -Research question informs the process of data collection and analysis. -Conclusion is clearly explained in providing recommendations based on the findings that limit healthcare professionals’ sensitivity and awareness of barriers to SAWLL.	Transparency: The purpose of the study is not clearly stated. Participant-driven inquiry: Participants did not shape the scope and breadth of questions, and participants were not involved in the analysis and interpretation of findings.	-SV not directly measured but inferred through qualitative narratives based on HCPs’ experiences who work with SAWLL. -Examples: HCPs silencing SAWLL; barriers rooted in social constructs (e.g., agism, sexism); interpersonal and institutional responses that prevent survivors from disclosing.
Greeson/2018 [33]	Level III (A)	-Purpose and aim are not clearly stated. -Research question is clearly stated: “What aspects of the local sociocultural community context do SART leaders believe influence the effectiveness of their SART?” and “How do SART leaders believe these aspects of the sociocultural context influence their SART’s ability to create an effective, coordinated response to SA?” (Greeson et al., 2018 [33], p. 447). -The phenomenon focused on SART coordination is clearly demonstrated. -Justification of the method is clearly explained as meaning is believed to be embedded in participants’ subjective experiences. -The study sample is representative of 169 interviews with SART members. -Researchers have experience and expertise in psychology and criminal justice. -Participant characteristics are described in terms of professional credentials and profession, gender, and active membership in a SART. -Sample size is adequate for reaching data saturation. -Processes of verification through member checking and confirmation with participants are not discussed. -Manual management of analysis is described. -Thematic findings are consistent with quotes of narrative data. -Research question informs process of data collection and analysis. -Conclusion is clearly explained in connection to the results. Researchers describe factors that interfere, while also facilitating an effective intervention through the use of SARTs in the treatment of survivors.	Diligence: No documentation of checking, confirming, and ensuring methodological coherence. Participant-driven results: SART leaders’ perceptions may not reflect the experiences of minoritized communities.	-SV not directly measured or operationalized as a construct; inferred from qualitative interviews with SART leaders. -Examples that may be interpreted as SV: victim blaming, questioning the legitimacy of reports, systems creating barriers due to denial, discomfort, or rejection of sexual assault as a legitimate issue.
Kelty/2018 [34]	Level III (A)	-The purpose of this study clearly focuses on assessing justice silos in Australia. -The aim is clearly stated as “to explore current forms of communication and practices and to identify if these interactions could be more effective in shielding four professional groups from the silo effect during the investigation and trial of homicide and SA matters” (Kelty et al., 2018 [34], p. 10). -Research questions are not clearly stated. -The phenomenon of justice silos is clearly stated. -Justification of the method is not clearly explained. -The sample is representative of 103 interviews with practitioners of forensic medicine, forensic science, law enforcement, and law. -Researchers have experience and expertise in law enforcement studies and forensic science. -Participant characteristics are clearly described in terms of age, region of Australia, and professional group. -Sample size is adequate for reaching data saturation. -Processes of verification through member checking and confirmation with participants are discussed. -Computer management analysis is described. -Thematic findings are consistent with quotes of narrative data. -Research question informs process of data collection and analysis. -Conclusion is clearly explained in connection to the results and delineated recommendations to address justice silos.	Transparency: Not clear whether one or more researchers analyzed each document and the process for formulating themes and categories. Delineates how information and decisions were made; however, limited information about how data were reviewed by others and how themes and categories were formulated. Self-reflection and self-scrutiny: No documentation of researcher considerations of how their personal experiences, background, or prejudices could bias or shape the analysis and interpretation. Disclosure statement and role of funding source acknowledge potential for bias; however, limited in-text self-reflection and self-scrutiny.	-SV recognized as an outcome from communication and practices in interactions with SA survivors. -SV inferred from the lack of collaboration among institutions and with SA survivors, which results in justice silos.
Kirkner/2021 [35]	Level III (A)	-The purpose of this study was to explore survivor and support provider recommendations in caring and responding to SA survivors. -This study aimed to examine recommendations in responding to SA survivors, -Research questions are not clearly stated. -The phenomenon is clearly stated as survivor-informed. -Justification of the method is clearly explained. -The study sample is representative as it includes 90 SA survivors and support providers separately, and 45 pairs of SA survivors and support providers. -Researchers have experience and expertise in criminology. -Participant characteristics are clearly described in terms of racial and ethnic background, socioeconomic status, education, employment, and relationship to support providers. -Sample size is adequate for reaching data saturation. -Processes of verification on how information was documented and how the feminist framework informed interviews, and transcript checks by the research team. The team discussed themes and patterns and developed a coding scheme; the researchers note that their analysis is “similar” to thematic analysis (p. 1011). Obtained information from multiple sources (survivors and their support providers). -Computer management analysis is described. -Thematic findings are consistent with quotes from narrative data. -Research question informs the process of data collection and analysis. -Conclusion is clearly explained in connection with the results to delineate practice and research implications.	Diligence: No documentation of checking, confirming, and ensuring methodological coherence.	-Actual and inferred experiences of SV of SA survivors based on SA survivors’ and support providers’ (paired together and separately) lived experiences. -Actual and inferred SV were described as separate, hurtful, and unresponsive responses that added to the trauma of SA. -Results of paired interviews with SA survivors and support providers, and separate SA survivor and support provider interviews demonstrate the impact of relational practices.
Munala/2022 [36]	Level III (A)	-Purpose and research question of the study are not clearly stated. -Aim of the study is clearly stated as to examine “the experiences and perspectives of health practitioners facing the challenges of providing services to female survivors of sexual violence” (Munala et al., 2022 [36], p. NP5294). -Phenomenon of focus is clearly stated. -Justification of the method is clearly described, as to how the ecological model and Colaizzi’s approach to analysis informed their decision-making, how data were reviewed, and how themes and categories were formulated. -Study sample of participants is representative, as 28 health practitioners from 8 post-rape care facilities in Nairobi, Kenya, were included. -Researchers have experience with the research area of public health in Kenya. -Participant characteristics are described in terms of gender, type of post-rape care facility, occupation, and experience working in a medical facility. -The sample size was adequate to achieve data saturation. -The process of verification in confirming and checking analysis and interpretation is well documented. -The manual method of analyzing the data is described. -Thematic findings consistent with the narrative data or quotes. -The findings are clearly connected to the research question, data collection, and analysis completed. -Conclusions are clearly explained.	Verification: did not include multiple sources to check or confirm findings. Participant-driven inquiry: Participants and/or community leaders were not included in triangulation; rather, triangulation was dependent on team analysis and use of ecological model.	-SV not directly operationalized but inferred from health practitioners’ experiences in post-rape care facilities and the outcomes witnessed from interactions in healthcare with SA survivors and their families. -Health practitioners’ experiences recall SA survivors’ fear of reporting due to interpersonal and individual consequences that harm SA survivors’ economic survival. -Informal reparations outside of systems, settled outside of systems effected by sociocultural and economic factors; depends on the knowledge of health practitioners and integration in the community of SA survivors served.
Munala/2018 [37]	Level III (A)	-Purpose and research question of the study are not clearly stated. -Aim of the study is clearly stated as to explore “the care of rape survivors from the perspective of healthcare practitioners” (Munala et al., 2018 [37], p. 217). -The phenomenon of focus is clearly stated as exploring healthcare practitioners’ mistrust of rape survivors. -Justification of the method is clearly described, as to The ecological model and Colaizzi’s approach to analysis. -Study sample of participants is representative, as 28 health practitioners from post-rape care facilities in Nairobi, Kenya, were included. -Researchers have experience with the research area of public health in Kenya. -Participant characteristics are described in terms of gender, type of post-rape care facility, occupation, and experience working in a medical facility. -The sample size was adequate to achieve data saturation. -The process of verification in confirming and checking the analysis is clearly described. -The manual method of analyzing the data is described. -Thematic findings consistent with the narrative data or quotes. -The findings are clearly addressed to the research question, and align with data collection and analysis processes. -Conclusions are clearly explained.	Verification: Lack of incorporation of multiple sources to check or confirm findings. Participant-driven inquiry: Participants and/or community leaders were not included in triangulation; rather, triangulation was dependent on team analysis and use of ecological model.	-SV is defined as “indirect method of assault that occurs through insensitive treatment of victims by individuals and institutions” (p. 218) but is not explicitly operationalized in the study. -Study focused on HCP perceptions, and the author came to the finding that HCPs’ doubt of SA survivors contributes to uncomprehensive and uncollaborative care that silences and harms SA survivors.
Patterson/2020 [38]	Level III (A)	-The purpose and aim of the study are clear. The aim is stated as to explore “if and how a national blended SAFE training influenced participants’ adoption of a patient-centered orientation” (Patterson et al., 2020 [38], p. 4757). -The research question is clearly presented as “How did the training help participants understand that their role extends far beyond the forensic exam to include a patient-centered approach that focuses on the well-being of survivors?” (Patterson et al., 2020 [38], p. 4761). -Justification of methods is not clearly described. -The phenomenon of focus is clearly stated to focus on shifting SAFE orientations to patient-centered care. -The study sample of participants is representative, as 64 SAFE participants participated in semi-structured interviews. -Researchers are experienced in social work. -Participant characteristics are described in terms of age, occupation, gender, education, and geographical environment. -The sample was adequate to achieve data saturation. -The process of verification in confirming and checking analysis and interpretation is clearly stated. -The manual method of analyzing the data is described. -Thematic findings consistent with the narrative data or quotes. -The findings are clearly connected to the research question, data collection, and analysis completed. -Conclusions are not clearly explained.	Diligence: The sample utilized may not be transferable across those who are SAFE-trained.	-SV not directly measured or operationalized; patient-centered orientation inferred to prevent SV of SA survivors based on SAFE participants’ experiences. -Interpretations of participants’ interviews suggest that rigid evidence collection, doubting SA reports, and non-therapeutic communication may contribute to SV of SA survivors.
Blain/2021 [39]	Level V (A)	-Purpose and case clearly stated as “how to balance providing a potentially beneficial intervention and avoiding re-traumatization and stigmatization” (Blain & Dombrowski, 2021 [39], p. 388). -A case report with commentary focused on healthcare clinician recommendations in practice for HIV pre-exposure prophylaxis (PrEP) or postexposure prophylaxis (PEP). -Findings of the case report are supported by relevance to TIC frameworks. -Practice recommendations clearly stated and informed by TIC frameworks in supporting decision-making and access to HIV PEP and PrEP following SA.	-Case report specific to TIC frameworks; lacks incorporation of relevant research. -Case report does not generate knowledge that is generalizable and transferable.	-SV not explicitly measured or operationalized. -Authors discuss the potential for SV and stigmatization in the clinical care of SA survivors, about pre-exposure prophylaxis (PrEP) or postexposure prophylaxis (PEP). -Authors delineate specific strategies to avoid SV that are population-specific to reduce risk but not supported by research, rather TIC framework implementation.
Cappelletti/2017 [40]	Level V (B)	-The purpose of the case report is clearly stated as “to report a case in which the forensic evaluation and identification of the SA were delayed because of…atypical and uncommon pattern of injury and the unconsciousness of the patient” (Cappelletti et al., 2017 [40], p. 258). -The case report clearly presents an 18-year-old woman initially admitted unconscious, presenting with injuries of a fourth-degree perineal laceration in nonconsensual fisting. -Case report is supported by relevant research on anal fisting injuries and treatment. -Recommendations are clearly stated; authors discuss the outcomes of this case with the need for medical and legal practices for treatment of perineal injuries and differential of consensual and nonconsensual fisting.	Conclusions: Draws fairly definitive conclusions based on the absence of research and recorded cases of treatment for perineal injuries related to fisting.	-SV not directly measured or operationalized. -The author recognizes inadequate identification and treatment of fourth-degree perineal lacerations due to SA may contribute to preventable poor health outcomes.
Klaver/2018 [41]	Level V (A)	-Purpose of the case report clearly presented as a case of a 25-year-old woman with severe delays in treatment complicated by positive HIV results, a rural setting, and tribal conflict involving SA. -Case report is clearly presented. -Findings of the case report are supported by relevant research on the impact of sociopolitical contexts on the availability of resources to support treatment of SA and antiretroviral therapy. -Recommendations are not clearly presented.	Recommendations are not clearly presented.	-SV not explicitly measured or operationalized. SV is inferred through a case in which sociopolitical economic context shapes institutional and cultural factors related to gender discrimination, resulting in severely delayed healthcare and added burden and harm to women.
Speck/2014 [42]	Level V (A)	-Purpose of the case report clearly presented as a case of an 18-year-old girl SA resulting in fourth-degree perineal lacerations. -Case report is clearly presented. -Findings of the case report are supported by relevant research on the limited research to inform treatment of severe injuries from fisting. -Recommendations clearly stated in connection to findings, including documentation of genital trauma using nuclear staining, anoscopy, and digital photography, and issues of consent with unconscious patients.	Unclear impact of surgical treatment from perspective of SA survivor.	-SV not directly measured or operationalized but inferred as surgical treatment with unconscious SA patient.
Auten/2015 [43]	Level II (B)	-Researchers identify what is known and not known of the effect of SANE programs on quality care, as well as potentials for simulation-based medical education to improve resident physicians’ comfort level and competence in providing care to SA victims. -The objective of this study is clearly stated as, “to develop and demonstrate the effectiveness of a low-fidelity hybrid simulation-based educational intervention that incorporated…well-defined tasks, appropriate level of difficulty, feedback in close proximity to task, and the ability to practice and improve in real time” (Auten, 2015 [43], p. 345). -Literature review of sources is mostly within the past five years. -Sample size is sufficient (*n* = 13 emergency medicine residents) with a cross-sectional study design for descriptive analysis. -The control group was not applicable for this study. -Data collection methods are clearly described as occurring over 11 months, during which residents received simulation training. A survey was distributed at 1 month pre-intervention and 3 months post-intervention of the simulation training. The study also included a critical action simulation examination and interview at completion of training. -Instruments of the questionnaire were not presented, including reliability and validity, which were not applicable. -Survey questions were presented, and evaluation criteria for the critical action examination were presented. -The response rate of the survey is not presented. -Results are presented clearly, showing that low-fidelity hybrid A simulation training improved emergency medicine residents’ comfort and competency in the treatment of SA survivors by 13%. -Fairly definitive conclusions, as low-fidelity hybrid simulation training is shown as a useful tool for training physicians on evidentiary exams and interviews considering privacy and direct treatment of SA survivors.	Sample size included 13 emergency medicine residents and is not able to be generalized. Unclear past training of emergency medicine residents may impact comfort and competence in providing care to SA victims. Study focuses on task-specifics for critical action simulation examination. Analysis and interpretation of findings of interview component completed with participants not presented.	-SV not explicitly measured or operationalized. -Risk of SV is inferred from emergency medicine residents’ self-reported feelings of comfort in managing SA survivors in questionnaires and interviews. -Potential for social desirability bias between examiner and interviewer with participating emergency medicine residents. -Task-specifics defined by dominant institutional perspective. -Competency described as task-specific and knowledge to ascertain effectiveness of simulation model.
Franchitto/2010 [44]	Level II (B)	-Research identifies what is known and not known of medical students’ need for knowledge related to forensic medicine and health law (Franchitto & Rougé, 2010 [44]). -The purpose of this study is unclear. -The literature included in this review was mostly from the past five years. -Sample size (*n* = 132 medical students) is sufficient in consideration of a cross-sectional design study to analyze survey data using demographic analysis, Pearson’s χ^2^ test, and Fisher’s test. -Control groups are not applicable. -Data collection methods are clearly described. -Instrument reliability and validity not applicable. -Survey response rate not presented. -The results were clearly presented of medical students’ experiences encountering medico-legal situations during years 2–6 of medical studies, activities medical students engaged in specific to forensic medicine and health law, and levels of competence and comfort level of using specialized knowledge in the area of forensic medicine and health law. -The content within the tables presented is consistent with the narrative. -Study limitations are identified and addressed in consideration of issues of representation of medical students broadly, as well as the lack of applicability across medical specialties of some items included in the questionnaire. -Conclusions are based on results that call for complementary initiatives to include partnership between clinical specialties.	The literature review is not comprehensive and mostly constrained to the last five years of the study. Results: Generalizability limited by convenience sample that may not be representative of medical students in other contexts outside of legal medicine in France. Relies on retrospective and subjective evaluations of students’ skills; objective measures of knowledge were not implemented. Conclusions: Fairly definitive conclusions related to HCP practices and education to prepare medical students to conduct examinations of SA victims in a legal medicine context.	-SV not directly measured or operationalized. -SV inferred by students’ evaluations of their skills to conduct SA examinations
Nathaniel/2021 [45]	Level III (B)	-The purpose is not clearly stated. -The research question is clearly stated to explore social workers’ views of intimate partner violence in Trinidad. -Justification of the method is clearly presented. -The phenomenon is clearly articulated. -Study sample participants were not representative of social workers. -Researchers are experts in behavioral sciences. -Participant characteristics are not described. -The sample size was not adequate to achieve data saturation. -Data analysis verification strategies were not used. -The method of data analysis is not described. -The narrative data of quotes aligns with the themes found. -The findings of this study align with data collection and analysis. -Conclusions clearly explained in considering social work interventions using a multi-systems approach.	Transparency: Lack of detail on purpose, sample size, and participant characteristics. Diligence: Details are not provided on the method or process for analyzing or interpreting data. Researchers do not discuss strategies for confirming interpretations across different sources. Verification: Different interview questions were asked among the women and men participants without providing a rationale or justification of the methodological approach.	-SV not explicitly measured or operationalized. -SV inferred through victim-blaming, societal norms that perpetuate GBV, and a lack of collaboration that contribute to SA survivors’ being unsupported and re-traumatized. These experiences erode trust of police, social workers, and the justice systems. -Social workers’ perceptions may affect networking and referrals, and survivors’ experiences with each agency.
Silva/2022 [46]	Level III (B)	-The clear purpose of this study is “to identify the perceptions of Primary Health Care workers regarding Violence Against Women (VAW)” (Silva et al., 2021 [46], p. 1). -The research question is not clearly stated. -Phenomenon focused on Primary Healthcare (PHC) workers’ perceptions of women accessing health centers. -Justification of the method is not provided. -The study sample is not representative of all PHC providers. The sample included 23 total PHC providers, including nurses, physicians, social workers, clinical psychologists, and neuropsychologist nurses. -Researchers demonstrate knowledge in the area of violence against women and PHC Health Centers in the context of Cape Verde, Africa. -Participant characteristics were described in terms of HCP role and gender. -Sample size adequate for reaching data saturation. -Verification process and method of analysis are not presented for data analysis. -Findings identified align with the narrative data (quotes). -Findings to address the research question align with data collected and the analysis process. -Conclusions are clearly explained in connection to the need to plan continuous education to improve PHC perceptions of VAW.	Transparency: The research question is not clearly presented. The justification of the method is not clear, although the use of the Bioecological Theory of Human Development to contextualize data is presented. Verification: The process of strategies to support trustworthiness (i.e., member checking, triangulation) is not presented. Insightful interpretation: Although the local context of PHC Health Centers in Praia, Ilha de Santiago of Cape Verde, Africa, is described in detail, the workforce of PHC Health Centers is not described in terms of HCP roles and/or gender.	-SV not explicitly measured or operationalized. -SV inferred as misconceptions of violence and victim blaming result in added harm to women seeking healthcare. -Reductionist views of violence are identified as narrowing HCP perceptions of VAW and the harm that results due to this perception shaping the care provided.
Strunk/2017 [47]	Level III (B)	-Purpose is clearly stated as “to evaluate pre-nursing and nursing students’ knowledge, attitudes, and beliefs about SA” (Strunk, 2017 [47], p. 70). -Comprehensive literature review within the past 5 years identifies gaps in knowledge. -No control group applicable. -Data collection clearly described, involving the distribution of a questionnaire in a large Midwestern university to pre-nursing students and Bachelor of Science Nursing students. -SA knowledge test, ATRV, and IRMA instruments used are reliable (Cronbach’s alpha ≥ 0.70). -Validity of instruments in questionnaire discussed: All three instruments are described in detail for content and aspects, with each instrument designed to minimize response bias. -The response rate to the questionnaire was greater than 25%, with a nearly 100% response rate. -Results presented clearly that last-semester nursing students tested lower for victim-blaming attitudes towards rape victims compared to pre-nursing students. -Three descriptive tables presented contain content consistent with the narrative. -Limitations of study identified and addressed: self-report survey using a convenience sample may affect students’ responses; cross-sectional design limits inferences of causality. -Conclusions based on results: Iterates the need for SA education in undergraduate nursing school through didactic and clinical experiences, as knowledge, attitudes, and beliefs can impact care provided to SA survivors.	Sample size: Sufficient for univariate and descriptive statistics. Results: Generalizability limited due to a convenience sample of predominantly white and female participants from one university. Conclusions: Cannot claim causality due to cross-sectional design and confounding of recent training for pre-nursing and bachelor’s nursing students. Self-report surveys that may be subject to social desirability bias. Conclusions: Definitive with limitations associated with study design.	-SV is not explicitly measured or operationalized. -Author uses the SA knowledge test, Illinois Rape Myth Acceptance Scale (IRMA), and Attitudes Toward Rape Victims Scale (ARVS) to provide insight into the current state of pre-nursing and nursing students’ knowledge, attitudes, and beliefs of SA in undergraduate education. -Authors discuss the influence of knowledge, attitudes, and beliefs on nurses’ behaviors that may contribute to SV, and the importance of understanding knowledge, attitudes, and beliefs to inform future education curriculum.
Bhagat/2018 [48]	Level III (C)	-Research identifies what is known and not known about the impact of guidelines and protocols on the treatment of survivors of sexual violence. -The aim is clearly stated as, “to assess awareness among doctors of the Ministry of Health and Family Welfare-Government of India March 2014 guidelines and protocols for medico-legal care of survivors of sexual violence” (Auten, 2015 [43], p. 118). -Literature review of sources is not mostly within the past five years. -Sample size is sufficient (*n* = 98 doctors in a medical college of Southern India) with a cross-sectional study design. -Control group was not applicable for this study. -Data collection methods are clearly described as a two-stage cluster sampling at a medical college in southern India across different departments using questionnaires. -Instruments of the questionnaire not presented; reliability and validity not applicable. -Response rate of questionnaire not presented. -Tables presented are consistent with narrative focused on presenting training material recommendations. -No limitations are identified or addressed. -Conclusions are based on results. The conclusion focuses on recommendations for implementing the training protocol to improve medical practitioners’ treatment of SA cases.	-Limited literature review to understand gaps in knowledge. -Instruments used in the questionnaire not mentioned. -Response rate of questionnaire not delineated. -No limitations are considered. -Descriptive conclusions based on this sample can be drawn to understand medical practitioners’ awareness of guidelines and protocols for sexual violence, but not generalized beyond the medical college in which the questionnaire was distributed.	-SV not directly measured using standardized instruments. -However, the authors discuss how a lack of awareness and training among medical practitioners regarding medico-legal guidelines can inadvertently contribute to the SV of SA survivors. -Authors provide recommendations to support the potential for the SV of SA survivors.

**Table 3:** Literature review matrix: secondary victimization.

Author (Year)	Aim/Purpose/Hypothesis	Research Design	Type of Healthcare Provider Interaction	Sample and Setting (Country)	Intervention	Measurements	Findings
Auten et al. (2015) [43]	To determine the effectiveness of a simulation-based SA response course for resident physicians at an institution without an on-site SANE program.	Quantitative	Resident physicians	12 emergency medicine residents (USA)	Low-fidelity hybrid simulation model.	Pre- and post-course Likert Scale questionnaires to measure comfort and competency to perform basic SA care.	The emergency medicine residents showed a 13% improvement in written examination scores pre- and post-intervention.
Bhagat, P. et al. (2018) [48]	To gain awareness of guidelines and protocols for SA cases.	Quantitative	Doctors	98 doctors at a medical college (India)	N/A	Questionnaire.	36.7% of doctors were unaware of guidelines and protocols of medico-legal care for SA cases.
Blain, M. & Dombrowski, J.C. (2021) [39]	To consider how to prevent re-traumatization and stigmatization while providing beneficial interventions.	Case study	Doctors, healthcare professionals	Case scenario of a patient who presents to a doctor following SA (Canada)	N/A	N/A	Healthcare providers should utilize trauma-informed care principles to aid in sharing decision making and support survivors’ autonomy when considering HIV prevention.
Cappelletti, S. et al. (2017) [40]	To present the clinical and morphological findings related to the sexual practice of fisting.	Case study	Lab, forensic examiner, medical doctor, forensic pathologist	Case of one 18-year-old with fourth-degree perineal laceration within vaginal and rectal mucosa (Italy).	N/A	Forensic SA examination, biological and toxicology analysis.	Forensic evaluation and the identification of the SA were delayed because of both the atypical and uncommon pattern of injury and the unconsciousness of the patient.
Franchitto, N., Rouge, D. (2010) [44]	To appraise the teaching of legal medicine among medical students.	Quantitative	Medical students	132 medical students enrolled at a medical faculty (France).	Pearson’s chi-square test and Fisher’s test.	Questionnaire.	92.4% of residents did not feel ready to examine victims of assault.
Goldblatt, H. et al. (2022) [32]	To explore barriers that fail to identify SA against women in late life.	Qualitative	Social workers, physicians, and nurses.	18 welfare and healthcare professionals (Israel).	Semi-structured interviews.	N/A	Leading negative emotions, lack of language, implications of cumulative and complex trauma, and social exclusion contribute to hindering identification of SA against women late in life.
Greeson, M.R. et al. (2018) [33]	To explore how sociocultural contexts may impact the effectiveness of SARTs.	Qualitative	Medical/forensic examiners/SANEs, medical, and advocacy systems.	169 leaders of SARTs (USA).	Semi-structured interviews.	N/A	A community’s sociocultural context consists of community-level factors that hinder or promote SARTs’ effectiveness.
Kelty, S.F. et al. (2018) [34]	To explore whether information could or should be shared between agencies while maintaining professional boundaries as legal experts.	Qualitative	Forensic physician/forensic nurse, forensic scientists, and pathologists.	121 practitioners from four professional groups that are involved in investigation/criminal proceedings of SA (Australia).	Semi-structured interviews.	N/A	Medical practitioners were semi-invisible in case decision-making, which may make their role/expertise unknown to other professions that collaborate with SA survivors.
Kirkner, A., Lorenz, K., & Ullman, S.E. (2021) [35]	To explore survivor and support provider recommendations for responding and caring for survivors.	Qualitative	Support providers.	1863 survivors and 45 survivors and support providers (USA).	Semi-structured interviews; questionnaire.	N/A	Recommendations from survivors to formal support providers and recommendations from support providers to support providers.
Klaver, R., Coe, J. (2018) [41]	To demonstrate lack of screening and treatment within facilities due to gender discrimination.	Case study	Medical doctor, HIV specialist.	Case of a woman with a 3-year history of increasing abdominal distension (Papua New Guinea).	N/A	N/A	Lack of healthcare resources and significant levels of gender discrimination led to grave complications. Also discuss PNG as the most dangerous place for women-IPV, SA, and thus increased risk for HIV.
Munala, L. et al. (2022) [36]	To better understand the vast care for a post-rape victim.	Qualitative	Clinical officers, nurses, trauma counselors, social workers, clinical psychologist, pharmacy technician, and health officer.	28 health practitioners (Kenya).	Semi-structured interviews.	N/A	Practitioners reported fear of consequences of reporting and seeking care, use of out-of-court settlements rather than intervention through formal health and criminal justice sector challenges, and attitudes toward sexual violence and survivors, normalization of rape, and victim-blaming attitudes.
Munala, L. et al. (2018) [37]	To explore the care of rape survivors from the perspective of healthcare practitioners.	Qualitative	Health practitioners.	28 health practitioners (Kenya).	Semi-structured interviews.	N/A	Practitioners perceived women seeking post-rape care as not genuine rape survivors. Attitudes towards CSWs influenced HCPs’ views and treatment of women.
Nathaniel (2021) [45]	To record views of intimate partner VAW which impacted practice to inform practice and education, and build knowledge bases.	Qualitative	Social workers.	Unspecified number of social workers (Trinidad).	Focus groups.	N/A	Victim behaviors contribute to victimhood, societal norms and attitudes contribute to intimate partner VAW, and inadequate responses from allied professionals contribute to intimate partner VAW.
Patterson, D. et al. (2020) [38]	To explore whether and how a national blended SAFE training influences participants’ adoption of a patient-centered orientation	Qualitative	SAFEs, healthcare providers.	64 healthcare professionals who received SAFE training (USA).	Semi-structured interviews.	N/A	Participants viewed training as helpful in creating the SAFE role as patient-centered healthcare. SAFE training reiterated patient-centered care, dispelled misconceptions about survivors, and delineated its impact on survivors’ well-being.
Silva, A. et al. (2022) [46]	To identify the perceptions of Primary Healthcare workers regarding VAW.	Qualitative	Health professionals.	23 health professionals (Cape Verde).	Semi-structured interviews.	N/A	Health professionals viewed VAW as restricted to physical violence, the result of financial dependence, and engaged in victim blaming.
Speck, P.M. et al. (2014) [42]	To show perpetrator schemes, traumatic reactions from victims of criminal sexual acts, and interventions to care for victims.	Case series	SAFE, ICU and ED nurses, urogynecologist, skilled nursing and assisted living home nurses.	9 cases of SA in older people (USA).	N/A	N/A	Nine cases are divided into dwellers in institutional and domestic settings. Institutional settings required enforcement of mandatory reporting.
Strunk, J. (2017) [47]	The purpose of this study was to evaluate pre-nursing and nursing students’ knowledge, attitudes, and beliefs about SA.	Quantitative cross-sectional, descriptive survey	Nursing students.	297 pre-nursing and nursing students (USA).	Univariate analysis of variance.	SA knowledge test, Illinois Rape Myth Acceptance Scale, and Attitudes Toward Rape Victims scale.	Rape myth acceptance is lower for nursing students in their last semester of college than in the pre-nursing group. Last semester nursing students held less victim-blaming attitudes toward rape victims than pre-nursing students.
Tien, L., Wu et al. (2017) [31]	To examine different perceptions of collaboration among team members and the related influences on collaboration.	Quantitative	Social workers, doctors, nurses, police officers.	140 team members (Taiwan).	Multivariate analysis of variance (MANOVA), multiple linear regression.	Index of Interdisciplinary Collaboration (IIC).	Collaboration by social workers was perceived significantly lower in the domains of interdependence and reflection on process. Professional roles, structural characteristics, personal characteristics, and the history of collaboration were positively associated with the overall perception of collaboration.

SA = sexual assault; SAFE = sexual assault forensic examiner; SART = sexual assault response team; VAW = violence against women.

**Table 4:** Results of studies.

Author/Year	Results
Tien/2017 [31]	-Overall score for social workers on interdependence was the lowest; social workers also showed lower reflection on process compared with health professionals. -Professional roles, structural characteristics, personal characteristics, and history of collaboration positively associated with overall perceived collaboration (R2 = 0.27, F(6, 134) = 9.43, *p* = 0.001).
Goldblatt/2022 [32]	-Four themes identified: (a) leading negative emotions, (b) lack of language, (c) implications of cumulative and complex trauma, and (d) social exclusion. -Professionals expressed their own negative dominant feelings in reaction to women’s disclosures. “I was quite disgusted by her descriptions”.
Greeson/2018 [33]	-Sociocultural community context either facilitated or hindered SARTs’ effectiveness. -Rape myths and victim blaming, denial of sexual assault happening here, discussion of sexual assault as taboo, male-dominated community, and everyone knows everyone were sociocultural characteristics of community contexts that cast sexual assault as an illegitimate issue.
Kelty/2018 [34]	-Four critical points identified in the process of investigating adult sexual assault that included the response of first responders, correct expertise, evidence submission and triage, and forensic analysis reports. -The response of first responders includes the risk of potentially losing valuable forensic evidence due to a lack of forensic knowledge or awareness by personnel responding. -The second critical point holds a risk for not having the correct expertise attending to the victim. -Timely submission and effective triage hold risk for losing valuable evidence and incorrect decisions being made about analysis and timeframe to collect evidence. -Three feedback loops identified that include suggesting a method to operationalize services provided; providing feedback to other disciplines in forensic specialties about the usability, clarity, and comprehension of reports; and developing practice improvement groups.
Kirkner/2021 [35]	-Recommendations delineated by recommendations from survivors to survivors, survivors to support providers, survivors to formal support sources, and support providers to support providers. -Ensure autonomy and affirmation of survivors to support individualized ways of coping. -Importance of self-care on behalf of providers recommended to other providers.
Munala/2022 [36]	-Cultural and economic factors identified as an aspect of understanding informal reparation and out-of-court settlements; “poverty plays a very big role in this”. -Patriarchal norms and behaviors placed blame on survivors.
Munala/2018 [37]	-Practitioners perceived women who accessed post-rape care as not true survivors of sexual violence or “genuine” rape survivors. -Practitioners believed that survivors who fell into a non-genuine category did so to access free antiretroviral medication.
Patterson/2020 [38]	-Continuous emphasis on patient-centered care, dispelling misconceptions of survivors, and explaining the impact of patient-centered care on survivors’ wellbeing all contributed towards influencing an orientation shift to patient-centered care. -Participants noted how patient-centered orientation improved their patient care but also made themselves more confident in their care.
Blain/2021 [39]	-To deliver care that is patient-centered, it is necessary to respond to patients’ requests and explain risks that inform patients’ ability to make informed decisions. -Avoiding harm to patients through re-traumatization and stigmatization is an ethical obligation. -A safe and trusting environment is required to facilitate collaboration between patient and provider.
Cappelletti/2017 [40]	-Forensic sexual assault examination focused on evidence collection. -Examination conducted on patient while unconscious and consent gathered from parents. -Biological and toxicological analysis facilitated per routine for HIV, gonococcus, and chlamydia.
Klaver/2018 [41]	-Limited resources available in rural areas led to delayed interventions, such as no blood bank, CT scan availability, HIV screening, and treatment facilities. -Violent tribal disputes in the highlands of PNG related to women, land, and pigs make accessing healthcare services challenging, and gender discrimination and gender-based violence are significant.
Speck/2014 [42]	-Nine case series divided into dwellers in institutional settings and domestic settings with community dwellers. -Institutional settings recommended to receive retraining on mandatory reporting of suspicion of sexual assault or abuse.
Auten/2015 [43]	-Mean pre-intervention written test score was 69.3%. -Comparing pre- and post-written test scores, there was an increase of 8.8%, with a standard deviation of 6.33 (*n* = 12). Representative of a 13.3% increase in overall examination scores (95% confidence interval 6.5–19.6%; *p* < 0.001). -7 of the 12 participants scored less than 80% on the posttest, and none scored more than 80% on the pretest.
Franchitto/2010 [44]	-Support materials available were not used by 70.4% of students (*n* = 93, 70.4%), and 20.5% did not know these materials existed. -At the end of receiving training, 95.6% did not feel ready to examine a corpse, and 92.4% did not feel ready to examine victims of assault.
Nathaniel/2021 [45]	-Three themes found among social workers: (1) victim behaviors contribute to victimhood; (2) societal norms and attitudes contribute to intimate partner violence against women; and (3) inadequate responses from allied professionals contribute to intimate partner violence against women
Silva/2022 [46]	-Participants perceived violence against women as physical abuse, which conditioned the recognition and intervention provided by primary healthcare providers. -Providers reported not always reporting cases of violence due to fear of being recognized or because women themselves were afraid of reporting.
Strunk/2017 [47]	-Pre-nursing students were most likely to demonstrate an acceptance of rape and rape myth beliefs (M = 86.6667, SD = 24.70854) compared to all other semesters. -Fourth-semester nursing students were least likely to accept rape and rape myth beliefs (M = 73.6833, SD = 23.24045) and attitudes that endorse rape and rape myth (M = 47.9, SD = 10.42926).
Bhagat/2018 [48]	-Doctors were most commonly not aware (36.73%) of new guidelines/protocols for medico-legal care for sexual assault victims, compared to 26.53% of doctors who were aware of new guidelines/protocols for the medico-legal care for sexual assault victims, and 23.47% of doctors who were partly aware. -On average, most doctors were partly aware of new guidelines/protocols for the physical examination of sexual assault survivors (35.72%). A total of 30.61% of doctors were aware of guidelines/protocols for the physical examination of sexual assault victims, and 20.40% were not aware.

## Data Availability

No new data were created or analyzed in this study.

## Supplementary Materials

The following supporting information can be downloaded at: https://www.mdpi.com/article/10.3390/healthcare14142111/s1; PRISMA 2020 Checklist.
