# Supplementary material for: A Systematic Review of Healthcare Providers’ Approaches to Practices That Contribute to Secondary Victimization of Sexual Assault Survivors
Source: Healthcare (Basel). 2026 Jul 14;14(14):2111. doi: 10.3390/healthcare14142111 (PMC13411014; doi:10.3390/healthcare14142111)
Supplement: Supplementary file 1 [file healthcare-14-02111-s001.zip › healthcare-4336682-supplementary.pdf]

PRISMA 2020 Checklist

Section and Topic	Item #	Checklist item	Location where item is reported
**TITLE**			
Title	1	Identify the report as a systematic review.	Page 1 of Main Manuscript
**ABSTRACT**			
Abstract	2	See the PRISMA 2020 for Abstracts checklist.	See Abstract
**INTRODUCTION**			
Rationale	3	Describe the rationale for the review in the context of existing knowledge.	Page 1 and 3-4 of Manuscript (Background)
Objectives	4	Provide an explicit statement of the objective(s) or question(s) the review addresses.	Page 1 and 3-4 of Manuscript (Background)
**METHODS**			
Eligibility criteria	5	Specify the inclusion and exclusion criteria for the review and how studies were grouped for the syntheses.	Page 4 and 5-6 (Inclusion and Exclusion Criteria) of Manuscript (Methods)
Information sources	6	Specify all databases, registers, websites, organisations, reference lists and other sources searched or consulted to identify studies. Specify the date when each source was last searched or consulted.	Page 4 of Manuscript (Methods)
Search strategy	7	Present the full search strategies for all databases, registers and websites, including any filters and limits used.	Page 4-5 (Search Strategy) of Manuscript (Methods)
Selection process	8	Specify the methods used to decide whether a study met the inclusion criteria of the review, including how many reviewers screened each record and each report retrieved, whether they worked independently, and if applicable, details of automation tools used in the process.	Page 6 (Study Selection) of Manuscript (Methods)
Data collection process	9	Specify the methods used to collect data from reports, including how many reviewers collected data from each report, whether they worked independently, any processes for obtaining or confirming data from study investigators, and if applicable, details of automation tools used in the process.	Page 4 and and page 13-14 (Data Extraction) Manuscript (Methods)
Data items	10a	List and define all outcomes for which data were sought. Specify whether all results that were compatible with each outcome domain in each study were sought (e.g. for all measures, time points, analyses), and if not, the methods used to decide which results to collect.	Page 8 (Data Extraction)

PRISMA 2020 Checklist

Section and Topic	Item #	Checklist item	Location where item is reported
			Manuscript (Methods)
	10b	List and define all other variables for which data were sought (e.g. participant and intervention characteristics, funding sources). Describe any assumptions made about any missing or unclear information.	Page 5-14 Manuscript (Methods), Page 30-31 Limitations
Study risk of bias assessment	11	Specify the methods used to assess risk of bias in the included studies, including details of the tool(s) used, how many reviewers assessed each study and whether they worked independently, and if applicable, details of automation tools used in the process.	Page 4-14 (Methods)
Effect measures	12	Specify for each outcome the effect measure(s) (e.g. risk ratio, mean difference) used in the synthesis or presentation of results.	Page 4 and 13-14 (Data Extraction) Manuscript (Methods)
Synthesis methods	13a	Describe the processes used to decide which studies were eligible for each synthesis (e.g. tabulating the study intervention characteristics and comparing against the planned groups for each synthesis (item #5)).	Page 4, 5-14 (Methods)
	13b	Describe any methods required to prepare the data for presentation or synthesis, such as handling of missing summary statistics, or data conversions.	Page 5, 5-14 Manuscript (Methods)
	13c	Describe any methods used to tabulate or visually display results of individual studies and syntheses.	Page 5-14 (Data Synthesis in Methods), Page 5 for Figure 1 (Search Strategy in Methods).
	13d	Describe any methods used to synthesize results and provide a rationale for the choice(s). If meta-analysis was performed, describe the model(s), method(s) to identify the presence and extent of statistical heterogeneity, and software package(s) used.	Page 10-14 (Table 1 in Data Synthesis of the Methods) and (Description of Studies); Table 2 in Results of Manuscript; (Table 3 of Study Quality and Risk Bias Appraisal in

Section and Topic	Item #	Checklist item	Location where item is reported
			Methods)
	13e	Describe any methods used to explore possible causes of heterogeneity among study results (e.g. subgroup analysis, meta-regression).	Page 14 (Data Synthesis in the Methods)
	13f	Describe any sensitivity analyses conducted to assess robustness of the synthesized results.	Page 14 (Data Synthesis in Methods)
Reporting bias assessment	14	Describe any methods used to assess risk of bias due to missing results in a synthesis (arising from reporting biases).	Page 6(Study Quality and Risk Bias of Individual Studies) in Methods . Page 27, Limitations.
Certainty assessment	15	Describe any methods used to assess certainty (or confidence) in the body of evidence for an outcome.	Page 10-14 (Study Quality and Risk Bias Appraisal) in Methods
**RESULTS**			
Study selection	16a	Describe the results of the search and selection process, from the number of records identified in the search to the number of studies included in the review, ideally using a flow diagram.	Page 14-18 (Description of Studies in Results) and Figure 1 of PRISMA flow diagram referenced on page 6
	16b	Cite studies that might appear to meet the inclusion criteria, but which were excluded, and explain why they were excluded.	Page 14
Study characteristics	17	Cite each included study and present its characteristics.	Page 14-18 for each theme presented in Results, as well as Table 1 and 2 of each study characteristics
Risk of bias in	18	Present assessments of risk of bias for each included study.	Page 18

Section and Topic	Item #	Checklist item	Location where item is reported
studies			under Study Quality and Risk Bias of Individual Studies (Table 2)
Results of individual studies	19	For all outcomes, present, for each study: (a) summary statistics for each group (where appropriate) and (b) an effect estimate and its precision (e.g. confidence/credible interval), ideally using structured tables or plots.	Page 14 under results, also includes Table 4 overviews results of each study
Results of syntheses	20a	For each synthesis, briefly summarise the characteristics and risk of bias among contributing studies.	Results under Study Quality and Risk Bias of Studies Synthesized Pages 18-19, Table 2
	20b	Present results of all statistical syntheses conducted. If meta-analysis was done, present for each the summary estimate and its precision (e.g. confidence/credible interval) and measures of statistical heterogeneity. If comparing groups, describe the direction of the effect.	Page 19 of Results under Summary of Findings presents all themes
	20c	Present results of all investigations of possible causes of heterogeneity among study results.	Results under Study Quality and Risk Bias of Studies Synthesized Pages 18-19, expanded upon in limitations
	20d	Present results of all sensitivity analyses conducted to assess the robustness of the synthesized results.	Results under Study Quality and Risk Bias of Studies Synthesized Pages 14-23
Reporting biases	21	Present assessments of risk of bias due to missing results (arising from reporting biases) for each synthesis assessed.	Results under Study Quality and Risk Bias

PRISMA 2020 Checklist

Section and Topic	Item #	Checklist item	Location where item is reported
			of Studies Synthesized Pages 18-23
Certainty of evidence	22	Present assessments of certainty (or confidence) in the body of evidence for each outcome assessed.	Results of themes presented on Page 15-23
**DISCUSSION**			
Discussion	23a	Provide a general interpretation of the results in the context of other evidence.	Page 23-26 of Discussion
	23b	Discuss any limitations of the evidence included in the review.	Page 27-28 of Limitations
	23c	Discuss any limitations of the review processes used.	Page 27-28 of limitations
	23d	Discuss implications of the results for practice, policy, and future research.	Page 26-27 of Implications to Practice
**OTHER INFORMATION**			
Registration and protocol	24a	Provide registration information for the review, including register name and registration number, or state that the review was not registered.	Page 4 first paragraph of Methods.
	24b	Indicate where the review protocol can be accessed, or state that a protocol was not prepared.	Page 4 first paragraph of Methods.
	24c	Describe and explain any amendments to information provided at registration or in the protocol.	Not applicable, no amendments made
Support	25	Describe sources of financial or non-financial support for the review, and the role of the funders or sponsors in the review.	Reported under funding portion of full manuscript materials for submission
Competing interests	26	Declare any competing interests of review authors.	No competing interests of review authors was reported under full manuscript materisl for

PRISMA

PRISMA 2020 Checklist

Section and Topic	Item #	Checklist item	Location where item is reported
			submission
Availability of data, code and other materials	27	Report which of the following are publicly available and where they can be found: template data collection forms; data extracted from included studies; data used for all analyses; analytic code; any other materials used in the review.	Page 4 of Methods for Template data collection forms, data extracted and included in studies, data used for all analysis, and other materials for review are reported in the paper (currently blinded for peer-review)

From: Page MJ, McKenzie JE, Bossuyt PM, Boutron I, Hoffmann TC, Mulrow CD, et al. The PRISMA 2020 statement: an updated guideline for reporting systematic reviews. BMJ 2021;372:n71. doi: 10.1136/bmj.n71.
This work is licensed under CC BY 4.0. To view a copy of this license, visit https://creativecommons.org/licenses/by/4.0/
